# Alterations in the Gut Microbial Composition and Diversity of Tibetan Sheep Infected With *Echinococcus granulosus*

**DOI:** 10.3389/fvets.2021.778789

**Published:** 2022-01-13

**Authors:** Zhigang Liu, Baishuang Yin

**Affiliations:** ^1^College of Life Science, Anqing Normal University, Anqing, China; ^2^Research Center of Aquatic Organism Conservation and Water Ecosystem Restoration in Anhui Province, Anqing Normal University, Anqing, China; ^3^Jilin Agricultural Science and Technology University, Key Lab of Preventive Veterinary Medicine in Jilin Province, Jilin, China

**Keywords:** gut microbiota, cystic echinococcosis, Tibetan sheep, *Echinococcus granulosus*, hydatidosis

## Abstract

Hydatidosis/cystic echinococcosis (CE) caused by *Echinococcus granulosus* is a parasitic zoonotic disease worldwide, threatening animal health and production and public health safety. However, it is still unclear that whether *E. granulosus* infection can result in the alteration of gut microbiota in Tibetan sheep. Therefore, a study was designed to investigate the influences of *E. granulosus* infection on gut microbiota of Tibetan sheep. A total of 10 ovine small intestinal contents (five from healthy and five from infected) were obtained and subjected to high-throughput sequencing by MiSeq platform. A total of 2,395,641 sequences and 585 operational taxonomic units (OTUs) were identified. *Firmicutes* and *Proteobacteria* were the most dominant phyla in all samples. Moreover, the proportions of *Armatimonadetes* and *Firmicutes* in the infected Tibetan sheep were significantly decreased, whereas *Actinobacteria, Chloroflexi*, and *Acidobacteria* had significantly increased. At the genus level, the *Christensenellaceae_*R-7_group and *Ruminococcaceae_*NK4A214_group were the predominant bacterial genera in all the samples. Furthermore, the healthy Tibetan sheep exhibited higher abundances of *Intestinimonas, Butyrivibrio, Pseudobutyrivibrio, Ruminococcaceae, Eubacterium_coprostanoligenes_*group, *Oxobacter, Prevotella_1, Ruminiclostridium_6, Coprococcus_1, Ruminococcus, Lachnospiraceae_UCG-002, Olsenella*, and *Acetitomaculum*, whereas *Kocuria, Clostridium_sensu_stricto_1, Slackia, Achromobacter*, and *Stenotrophomonas* levels were lower. In conclusion, our results conveyed an information that *E. granulosus* infection may cause an increase in pathogenic bacteria and a decrease in beneficial bacteria. Additionally, a significant dynamical change in gut microbiota could be associated with *E. granulosus* infection.

## Introduction

Tibetan sheep is an ancient species of the Qinghai-Tibet plateau that prevails from central Kazakhstan to Shanxi province in China and from Altai mountains to Himalaya. It is the largest prevailing wild-type sheep in the world and has adapted to hypoxic conditions (3,500–5,000 m above sea level) and low temperature of the area (1). This sheep is a primary source of income, leather, milk, and meat for the local herdsmen (2). The abundant herbage resources in the Tibetan plateau have provided subsistence conditions for this sheep. However, this region has a higher incidence of echinococcosis in sheep and their herdsmen probably due to prevailing substandard hygienic practices (3).

Hydatidosis/cystic echinococcosis (CE) is a worldwide zoonosis caused by *Echinococcus granulosus sensu lato* that causes health and economic losses, especially in the areas of Central Asia, western China, southern Europe, North and Central Africa, and south-western Latin America (4). In the People's Republic of China, it is mainly prevalent in the western parts of the country including Xinjiang, Qinghai, Gansu, Tibet, and Sichuan provinces (5). In pastoral areas, the human infection rate can reach 50% (6). *Echinococcus granulosus* mainly infects the liver but may also infect the lungs, heart, brain, and intestines in the hosts, resulting in rashes, fever, abdominal pain, diarrhea, and even death (5).

The intestine colonizes a great variety of microbes including bacteria, protozoa, and fungi (7). Gut microbiota plays important roles in metabolism, nutrient absorption, and mucosal immunity (8). The variation in the normal gut microbiota can influence metabolic activities and health of the host (9). The composition of gut microbiota is influenced by several extrinsic and intrinsic factors, including food type, environment, species, age, and disease (10). Therefore, the richness and diversity of gut microbiota can indicate host health status and indirectly of various host disease situations. *E. granulosus* can inhabit the small intestine and liver of the host. However, little is known about the characteristics of gut microbiota in Tibetan sheep infected with *E. granulosus*. Therefore, the objective of the present study was to compare and analyze the differences in gut microbiota in healthy and *E. granulosus*-infected sheep.

## Materials and Methods

### Sample Acquisition

A total of 10 (five healthy and five *E. granulosus*-infected) 1-year-old Tibetan sheep were selected from a commercial feedlot farm at Tibet, China. The infected sheep was diagnosed by a professional veterinarian and determined by molecular biology. The ratio of females to males in both groups was 2:3. The selected Tibetan sheep were fed on free-range grassland and self-propagated *via* the commercial farm. All the selected sheep possessed a similar genetic background, and no other disease was observed prior to the sample collection. All the sheep were euthanized, and the contents were obtained from intermediate areas of the duodenum, ileum, and jejunum of the control and the infected groups. The collected intestinal contents were transported immediately in sterile plastic bags and stored at −80°C until further analysis. Moreover, the diseased liver and lungs were collected for microscopy and DNA extraction.

### DNA Extraction and PCR Amplification of *E. granulosus*

For molecular confirmation, the total genomic DNA of *E. granulosus* was isolated using the TIANamp Genomic DNA Kit according to the manufacturer's instructions. Moreover, the specific primers (forward: 5′-ATTATAGAAAATTTTCGTTTTACACGC-3′ and reverse: 5′-AAGCATGATGCAAAAGGCAAATAAACC-3′) were synthesized to amplify the fragment of the cox1 of mitochondrial gene. The design of the primer was based on previous research and synthesized by Jinsirui Biotechnology Co., Ltd. (Nanjing, China) (11). The PCR-amplified products were analyzed through 1.5% agarose gel by following electrophoresis and the Hi-TIANgel Midi Purification Kit. Subsequently, the PCR products were delivered to the Qingke Biotech Company (Wuhan, China) for sequencing analysis and subjected to BLAST in NCBI. Based on sequencing results, a phylogenetic tree was developed by using MEGA 7 software to determine the conformation of parasitic species.

### Microbial Genomic DNA Extraction

DNA of each sample was extracted using QIAamp DNA Mini Kit following the manufacturer's instructions. To assess the extraction quality of DNA, 0.8% (*w*/*v*) agarose gel electrophoresis was used. Moreover, the concentration of the DNA was quantified by using a NanoDrop^TM^ spectrophotometer.

### Amplification and Sequencing of 16S rRNA Gene

Specific gene primers (338F: ACTCCTACGGGAGGCAGCA and 806R: GGACTACHVGGGTWTCTAAT) were produced. PCR amplification products were assessed through gel electrophoresis. AxyPrep DNA Gel Extraction Kit was used to recycle target fragment. The Quant-iT PicoGreen dsDNA Assay Kit (Invitrogen, Massachusetts, USA) was used for fluorescent quantitation of PCR amplification recovery products on a microplate reader according to preliminary quantitative results of electrophoresis. Sequencing libraries were constructed using TruSeq Nano DNA LT Library Prep Kit (Illumina, USA) following the manufacturer's specification. The End Repair Mix2 was used to repair the sequence ends of the amplified products. A magnetic bead screening system was used to remove the self-connected fragments in the linker followed by the purification of the library system. PCR was done to amplify the obtained DNA fragments for enriching sequence library templates. The AMPure XP Beads were used to repurify the enriched library product.

Before sequencing, the quality of libraries was detected on Agilent Bioanalyzer, and the qualified libraries should only have one peak and no linker. Moreover, the libraries were quantified via using Quant-iT™ PicoGreen™ dsDNA Assay Kit, and library concentrations above 2 nM were finally selected. The selected sequencing library was diluted by gradient and mixed in proportion. The mixed libraries were subjected to 2 × 250-bp paired-end sequencing using MiSeq Reagent Kit V3 (600 cycles) on the MiSeq sequencing machine.

### Statistical and Bioinformatics Analysis

The original 16S rRNA data files were subjected to initial quality screen and formal analysis by QIIME™ software (version 1.9.1). Also, short and low-quality sequences (<200 bp) were removed. The clustering program VSEARCH (1.9.6.) was used to merge the sequences and partition operational taxonomic unit (OTU) at ≥97% sequence similarity. A confidence threshold of 0.8 was used to generate a sequence of each OTU as per the Ribosomal Database Project (RDP). Furthermore, the MUSCLE software was used for multiple sequence alignments and phylogenetic analysis of different OTUs. The sparse curves and four diversity indexes (Chao1, ACE, Simpson, and Shannon) were used for assessing sequencing depth and alpha diversity, respectively. R (v3.0.3) and GraphPad Prism (version 6.0c) were used to statistically analyze the data. A value of *p* ≤ 0.05 was considered statistically significant. The standard deviation values were presented as means ± SD.

## Results

### Clinical and Molecular Examination

**Figure 1:**
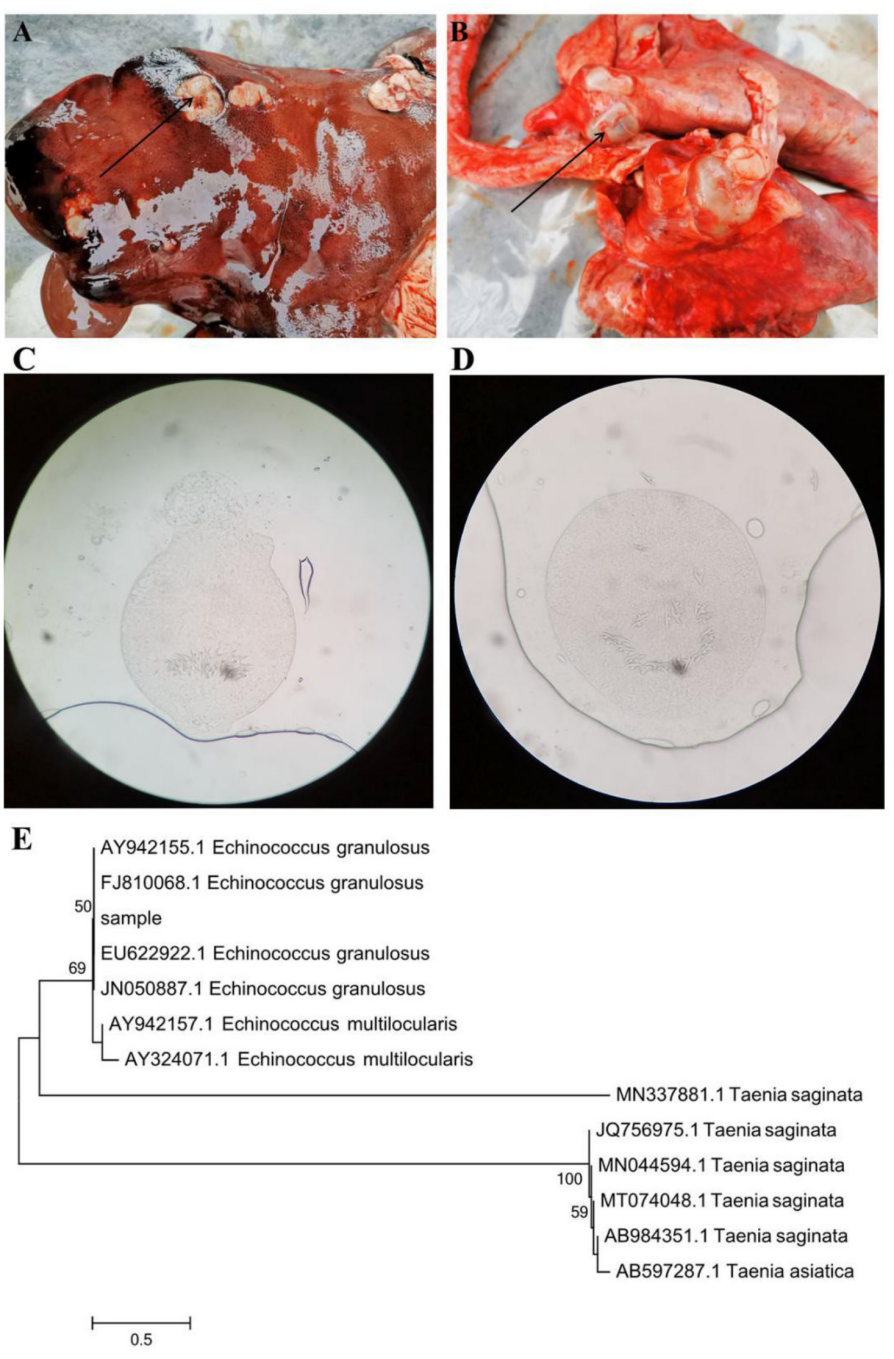
Gross examination of the liver **(A)** and lung **(B)** in the hydatid-infected Tibetan sheep. The black arrows indicate hydatid sacs. Microscopic observation and phylogenetic analysis. **(C,D)** Morphological observation of *Echinococcus granulosus*. **(E)** A phylogenetic tree constructed by using the neighbor-joining method.

The visual assessment revealed that the healthy Tibetan sheep possessed an active mental state and appetite. Conversely, the infected sheep showed dispiritedness, decreased appetite, and dyspnea. The infected sheep also showed signs of pain and dodge when touched on the abdomen. Moreover, hydatid sacs in the liver and lungs were observed in the infected sheep on postmortem examination (Figures 1A,B). Additionally, we also observed obvious parasite morphology through the microscope (Figures 1C,D). Phylogenetic analysis of collected samples revealed high homology with the *E. granulosus* under the statistical evaluation assessed using 1,000 bootstraps values (Figure 1E).

### Sequence Analyses

**Figure 2:**
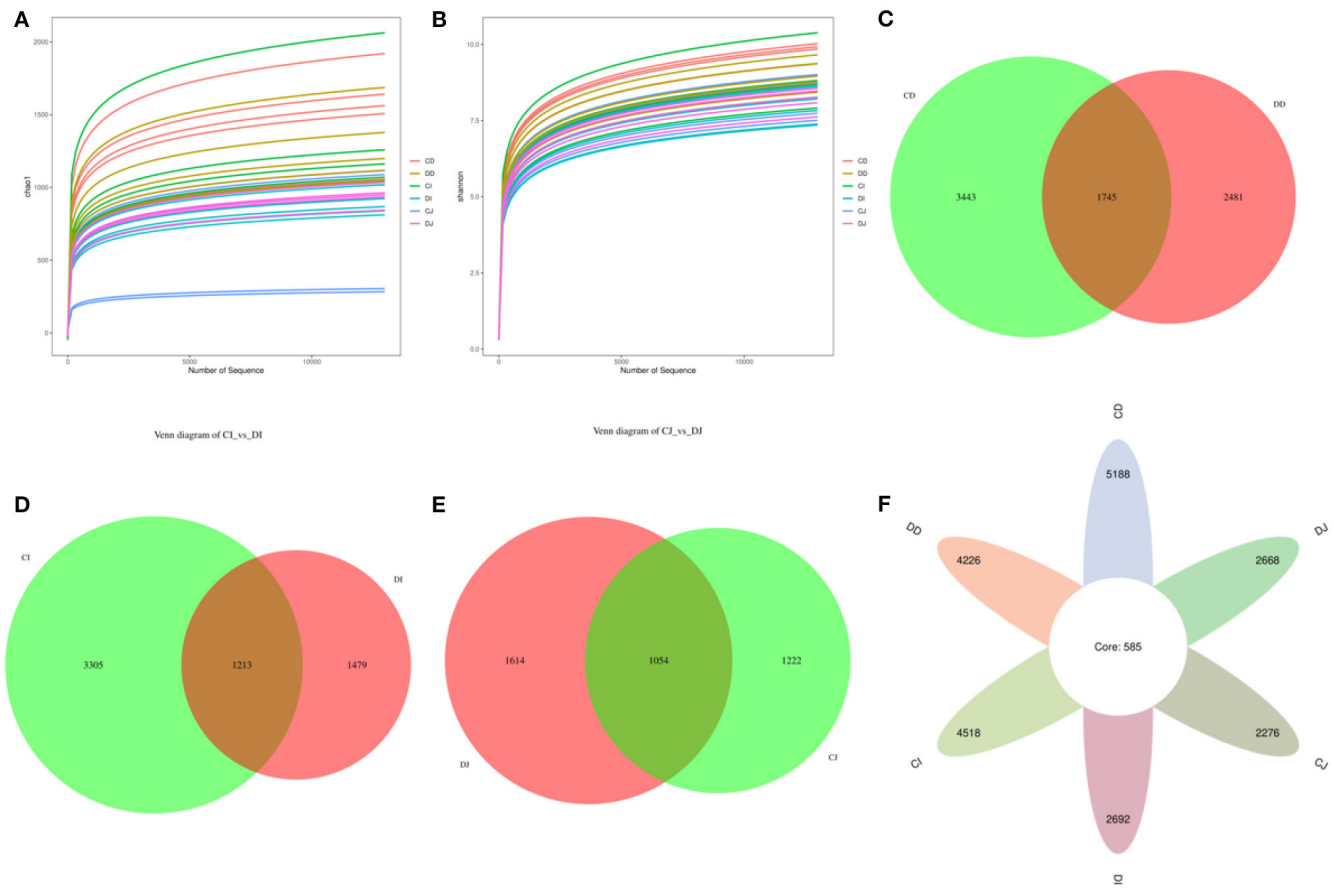
Feasibility analysis and venn diagrams. The rarefaction curves **(A,B)** were used to evaluate the adequacy of sequencing for each sample. Each curve indicates a sample. **(C)** Venn diagrams of the OUTs distribution in the CD and DD. **(D)** Venn diagrams of the OUTs distribution in the CI and DI. **(E)** Venn diagrams of the OUTs distribution in the CJ and DJ. **(F)** Venn diagrams for core OTUs compositions.

In this study, a total of 418,913, 423,714, 419,745, 422,034, 425,070, and 417,234 raw sequences were acquired from CD (control duodenum), DD (*E. granulosus*-infected duodenum), CI (control ileum), DI (*E. granulosus*-infected ileum), CJ (control jejunum), and DJ (*E. granulosus*-infected jejunum), respectively (Table 1). After optimizing the original data, 2,395,641 valid sequences were acquired from all the samples (Table 1). Moreover, the rarefaction curve (Shannon and Chao1 curves) for all samples extended all the way to the right end of the *x*-axis, indicating that the present sequencing depth was sufficient to reflect the diversity of microorganisms contained in all groups (Figures 2A,B). Following taxonomic assignment, a total of 21,568 OTUs (CD = 5,188, DD = 4,226, CI = 4,518, DI = 2,692, CJ = 2,668, and DJ = 2,276) were recognized, and 585 OTUs were common in all the samples (Figures 2C–F). Furthermore, the quantity of unique OTUs in the CD, DD, CI, DI, CJ, and DJ was 3,443, 2,481, 3,305, 1,479, 1,222, and 1,614, respectively (Figures 2C–E).

**Table 1:** The sequence information of each sample.

**Sample**	**Raw_reads**	**Clean_Reads**	**Effective (%)**
CD1	87,329	83,979	96.16
CD2	81,317	78,570	96.62
CD3	85,026	82,901	97.50
CD4	80,419	76,727	95.41
CD5	84,822	83,092	97.96
DD1	84,356	81,842	97.02
DD2	86,148	83,359	96.76
DD3	85,099	81,681	95.98
DD4	87,327	81,985	93.88
DD5	80,784	77,660	96.13
CI1	81,942	79,400	96.90
CI2	85,437	80,773	94.54
CI3	81,167	76,745	94.55
CI4	86,694	84,025	96.92
CI5	84,505	79,959	94.62
DI1	86,104	74,026	85.97
DI2	83,586	80,690	96.54
DI3	84,685	82,738	97.70
DI4	81,448	79,968	98.18
DI5	86,211	84,695	98.24
CJ1	86,891	80,587	92.74
CJ2	87,620	82,400	94.04
CJ3	84,176	74,020	87.93
CJ4	81,333	78,002	95.90
CJ5	85,050	81,878	96.27
DJ1	84,331	80,138	95.03
DJ2	82,550	73,195	88.67
DJ3	87,121	81,116	93.11
DJ4	82,436	71,938	87.27
DJ5	80,796	77,552	95.98

*CD, control duodenum; DD, diseased duodenum; CI, control ileum; DI, diseased ileum; CJ, control jejunum; DJ, diseased jejunum*.

### Alterations in the Gut Microbial Diversities

**Figure 3:**
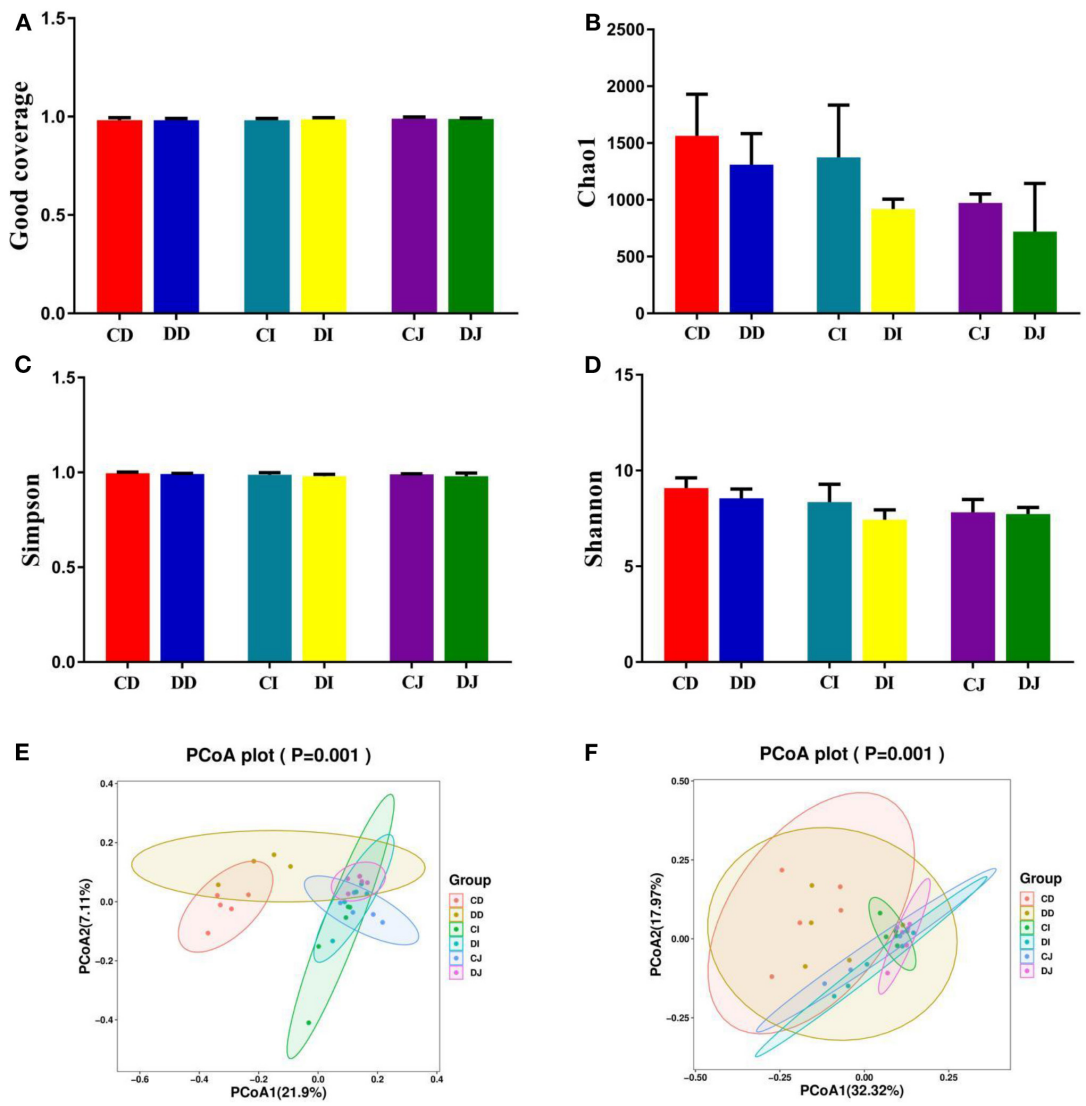
The differences of gut microbial diversities between healthy and hydatid-infected Tibetan sheep were non-significant. The alpha diversity of intestinal microbial community can be evaluated by the **(A)** Good's coverage, **(B)** Chao1, **(C)** Simpson, and **(D)** Shannon. **(E)** PCoA map based on weighted uniFrac distance **(F)** PCoA map based on unweighted uniFrac distance.

In the present study, Good's coverage estimates were approximately 100% for all the samples, exhibiting excellent coverage (Figure 3A). The average of Chao1 index in the control (CD, CJ, and CI) and *E. granulosus*-infected (DD, DJ, and DI) groups varied from 972.40 to 1,563.00 and 719.60 to 1,308.60, respectively (Figure 3B). Moreover, the duodenum possessed the highest Chao1 and Shannon indices as compared to the jejunum and ileum. The average of Chao1 indices in CD, CI, and CJ groups (1,563.00, 1,374.00, and 972.40, respectively) was higher than that in DD, DI, and DJ groups (1,308.60, 918.40, and 719.60, respectively), and a statistically non-significant difference (*P* > 0.05) was found between these groups (Figure 3B). The gut microbial abundance values did not differ significantly between the control and *E. granulosus*-infected groups by Chao1 index. Similarly, the average of Simpson and Shannon indices of the control group was higher than that of the *E. granulosus*-infected group, whereas no obvious difference was found between the two groups (Figures 3C,D). A non-significant difference (*P* > 0.05) in the gut microbial evenness was found in the *E. granulosus-*infected and control groups. PCoA plots, which reflect the difference and similarity between groups and individuals, were generated to assess the gut bacterial beta diversity. The beta diversity analysis indicated that the individuals in all groups were clustered together, suggesting that the differences in the principal compositions of gut microbial community of the different groups were insignificant (Figures 3E,F).

### Changes in the Composition of Gut Bacterial Community

**Figure 4:**
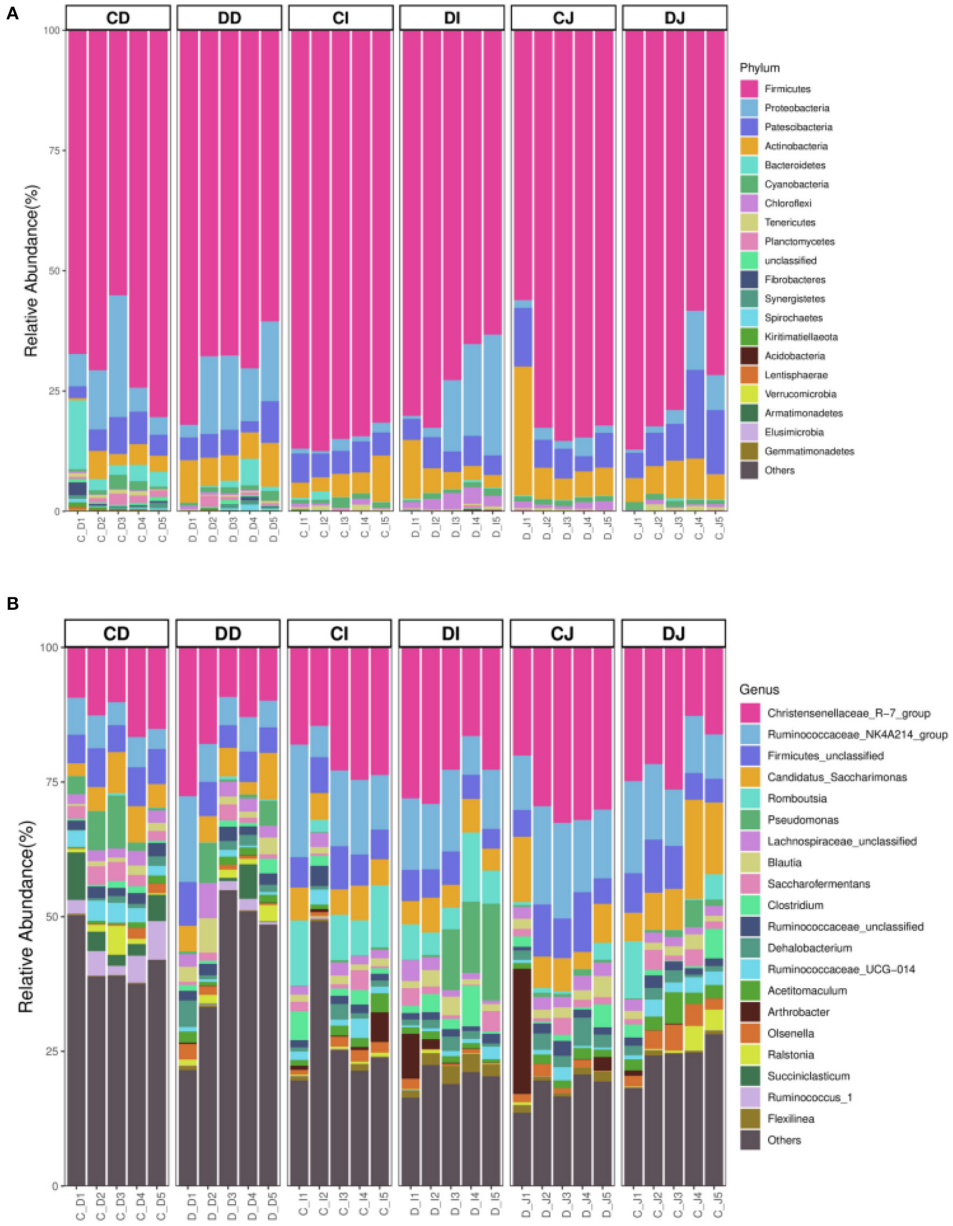
The relative richness of the gut microbiota in healthy and Echinococcus granulosus infected Tibetan sheep. **(A)** The top 20 dominant phylum of the Tibetan-sheep gut microbiota. **(B)** The top 20 major genera of the Tibetan-sheep gut microbiota.

**Figure 5:**
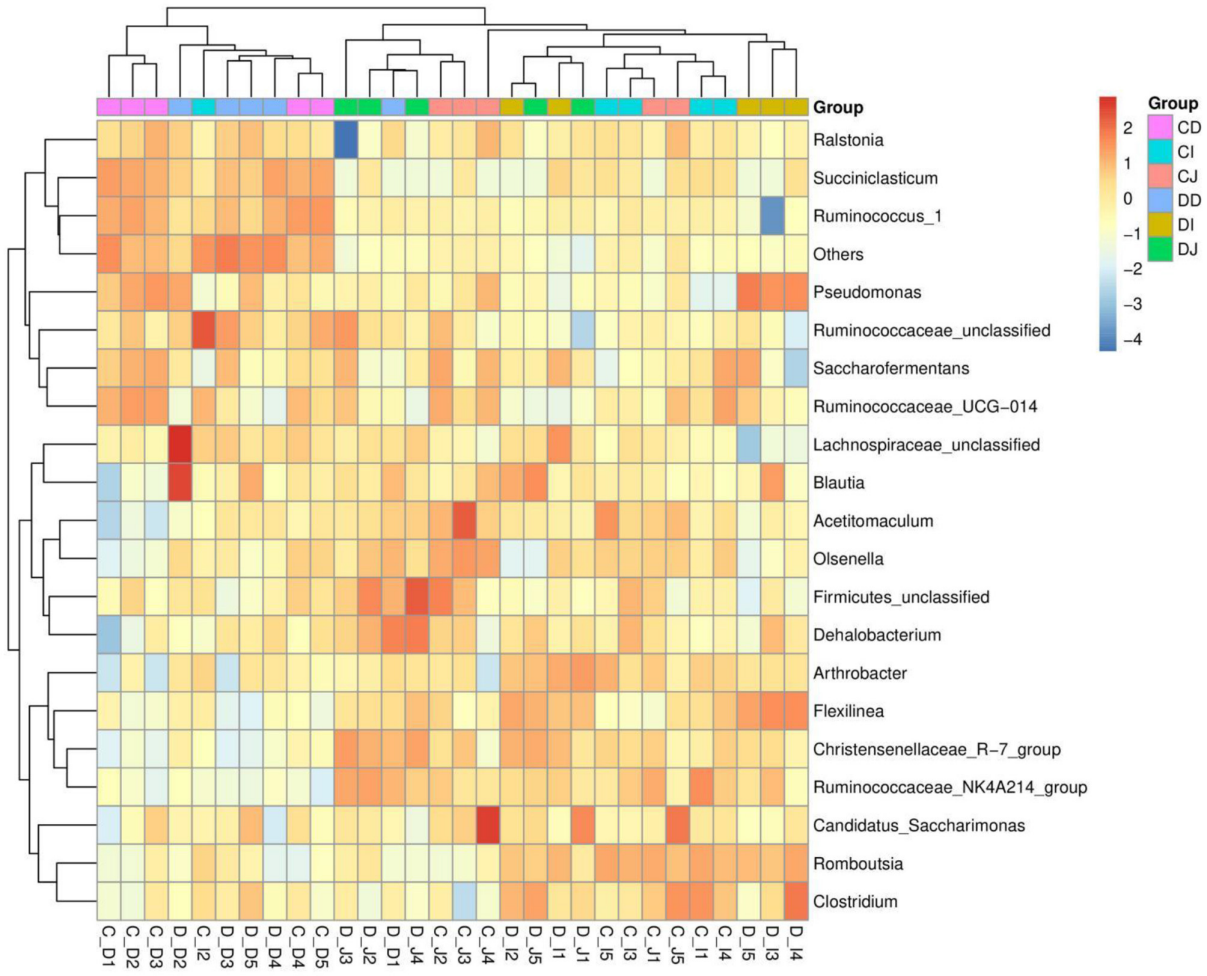
Heatmap of the dominant genera in different groups.

The composition and structure of gut microbiota in various intestinal segments (duodenum, jejunum, and ileum) were analyzed at different taxonomical levels, respectively (Figure 4). At the phylum level, *Firmicutes* (69.54, 69.63%) and *Proteobacteria* (10.60, 12.35%) were dominant in the duodenum of CD and DD groups, and the sum of abundances was more than 80% (Figure 4A). In the CI, DI, CJ, and DJ groups, the most significant bacteria at phylum level were *Firmicutes* (85.07, 72.79, 75.67, and 78.17%), *Patescibacteria* (5.41, 5.09, 10.32, and 6.92%), *Proteobacteria* (1.41, 12.32, 4.89, and 2.22%), and *Actinobacteria* (4.96, 5.28, 6.42, and 9.77%) (Figure 4A). *Christensenellaceae_R-7_group* (12.78, 15.51, 20.34, and 28.84%), *Ruminococcaceae_NK4A214_group* (5.31, 7.91, 12.08, and 14.45%), *Firmicutes_unclassified* (6.25, 5.17, 6.91, and 7.58%), and *Candidatus_Saccharimonas* (5.12, 5.17, 10.30, and 6.90%) were the four most dominant genera in the CD, DD, CJ, and DJ groups (Figure 4B). Moreover, the most abundant genera were *Christensenellaceae_R-7_group* (20.74%), *Ruminococcaceae_NK4A214_group* (12.95%), *Firmicutes_unclassified* (6.33%), and *Romboutsia* (8.16%) in the CI group, while *Ruminococcaceae_NK4A214_group* (23.78%), *Firmicutes_unclassified* (11.75%), *Romboutsia* (6.74%), and *Pseudomonas (*8.59%) were observed as predominant in the DI groups. Interestingly, *Christensenellaceae_R-7_group* was constantly the most preponderant bacterium in all the samples. Moreover, the primary composition of gut bacterial community in different intestinal samples could also be found in the heatmap (Figure 5).

**Figure 6:**
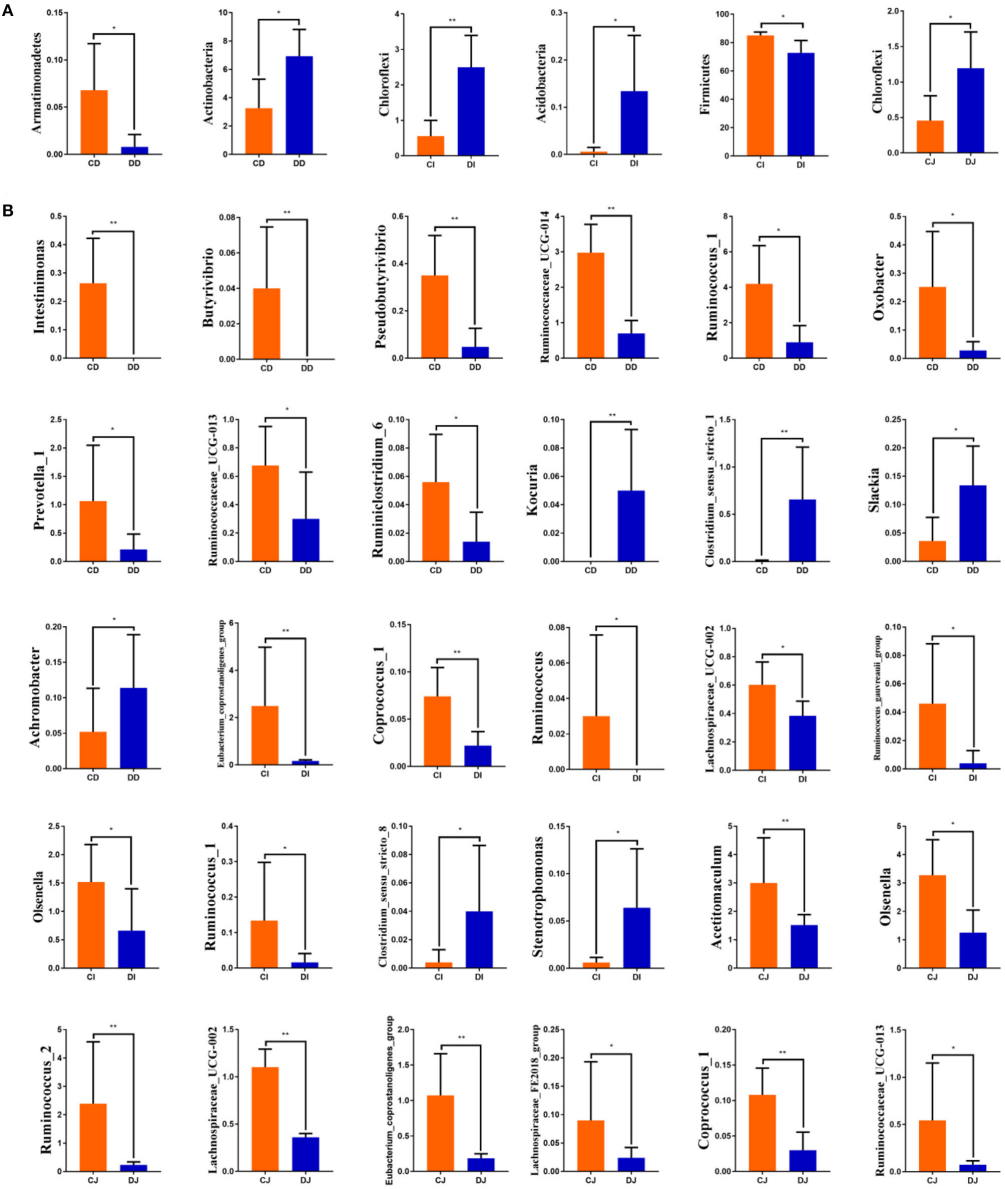
Significant changes in the compositions of gut microbial community at the phylum **(A)** and genus **(B)** levels.

The comparison of gut microbiota between the control (CD, CI, and CJ) and *E. granulosus*-infected (DD, DI, and DJ) groups indicated that the abundance of *Armatimonadetes* at the phylum level in the CD group was distinctly higher than in the DD group, while the *Actinobacteria* content was lower (*P* < 0.05) (Figure 6A). *Chloroflexi* and *Acidobacteria* in the CI group were distinctly lower than in the DI group, whereas the *Firmicutes* level was higher (*P* < 0.05 or *P* < 0.01) (Figure 6A). Furthermore, the relative abundance of *Chloroflexi* was significantly more dominant in the DJ group than the CJ group (*P* < 0.05) (Figure 6A). At the genus level, *Intestinimonas, Butyrivibrio, Pseudobutyrivibrio, Ruminococcaceae_UCG-014, Ruminococcus_1, Oxobacter, Prevotella_1, Ruminococcaceae_UCG-013*, and *Ruminiclostridium_6* were distinctly higher in the CD group (*P* < 0.05) than in the DD group, while the *Kocuria, Clostridium_sensu_stricto_1, Slackia*, and *Achromobacter* levels were lower (*P* < 0.05 or *P* < 0.01) (Figure 6B). Meanwhile, *Eubacterium_coprostanoligenes_group, Coprococcus_1, Ruminococcus, Lachnospiraceae_UCG-002, Ruminococcus_gauvreauii_group, Olsenella*, and *Ruminococcus_1* were significantly higher in the CI group than in the DI group, while the *Clostridium_sensu_stricto_8* and *Stenotrophomonas* contents were lower (*P* < 0.05) (Figure 6B). Furthermore, a comparison of the CI and DI groups displayed a significant increase (*P* < 0.05) in the abundance of *Acetitomaculum, Olsenella, Ruminococcus_2, Lachnospiraceae_UCG-002, Eubacterium_coprostanoligenes_group, Lachnospiraceae_FE2018_group, Coprococcus_1*, and *Ruminococcaceae_UCG-013* (*P* < 0.05) (Figure 6B).

## Discussion

Gut microbial community is a dynamic and complicated system that significantly influences the host physiology (12). Moreover, increasing evidence indicated that gut microbial community poses a barrier for the host against colonization and invasion of the pathogenic bacterium (13, 14). Therefore, the analysis and investigation of gut microbiota possess significance in preventing and treating certain diseases. To date, numerous research have investigated the relationship of microbial community structure and multiple diseases including diarrhea, diabetes, asthma, and obesity (9, 10). However, to our best understanding, only one study focused on the impact of *E. granulosus* infection on gut microbiota (15). In this study, we analyzed the intestinal microbiota composition in healthy and infected Tibetan sheep by high-throughput sequencing techniques.

Although, most of the research on gut microbiota employs fecal samples, the diversity of gut microbial community cannot be completely reflected by these samples (12). Therefore, intestinal samples were collected to evaluate the changes of gut microbiota. We observed that the number of OTUs and the alpha diversity indexes were lower in the *E. granulosus*-infected group than the control group, indicating that *E. granulosus* infection caused a downward trend in the abundance and diversity of gut microbiota. Similarly, He et al. also indicated that parasitic infection decreased the diversity of gut microbiota in piglets (16). The gut microbiota is an important barrier for the host against the invasion of pathogenic bacteria, which in turn depends on the normal gut microbial composition and diversity (17). Therefore, the lower diversity and richness of gut microbiota could increase the risk of infections caused by secondary pathogenic bacteria (14). Numerous studies revealed that the intestinal function was positively related to gut microbial abundance, and the higher gut microbial richness and diversity favor nutrient absorption and conducting complex physiological functions (18, 19). It has been demonstrated that parasites can cause weight loss and malnutrition of the host by affecting the intestinal absorption function (20). Therefore, the influence of parasitic infection on intestinal functions may be mediated by affecting gut microbial composition and structure.

The gut microbial community is a special ecosystem in the intestine consisting of various microbes that interact as commensals, pathogens, and/or opportunistic pathogens (21, 22). The interaction among various types of bacteria not only promotes metabolism and nutrient absorption but also contributes to the immune system maturation against infection, hence decreasing the risk of disease (23). Generally, the composition of gut microbiota in ruminants is affected by multiple factors, such as host age, nutrition, sex, stress, disease, and growing environment (24). This study revealed that *Firmicutes* and *Proteobacteria* were the dominant phyla in all the samples, regardless of the health status. Moreover, those phyla were also observed to be widely distributed in goats, yak, and cattle, indicating their importance in intestinal ecology and function (25, 26). Interestingly, although *E. granulosus* infection cannot alter the diversity of dominant bacterial phyla in Tibetan sheep, the percentage of some bacteria was altered dramatically. Compared with the CI group, the ratio of *Proteobacteria* and *Actinobacteria* in the gut microbiota of the DI group was increased, while the proportion of *Firmicutes* was decreased. It is known that *Firmicutes* mainly consists of many gram-positive bacteria including *Lactococcus, Listeria, Bacillus*, and *Lactobacillus* (27). Previous research has shown that *Firmicutes* plays a key role in the digestion of proteins and carbohydrates (28). Therefore, the abundance of *Firmicutes* in the gut environment is conducive to meet the energy and nutritional demands in animals (29). Furthermore, *Lactobacillus, Listeria*, and *Lactococcus* in the *Firmicutes* are considered as beneficial bacteria, which play their roles in maintaining gut flora balance and preventing pathogenic invasion (30, 31). *Proteobacteria* comprises great amounts of (gram-negative) pathogenic bacteria, e.g., *Salmonella, Helicobacter pylori, Vibrio cholera*, and *Escherichia coli*, and is the largest phylum (32, 33). The abovementioned pathogenic bacteria cause vomiting, diarrhea, gastritis, gastrointestinal ulcers, and even death, which seriously threaten the health of animals (34). Consequently, the higher percentage of *Proteobacteria* in the microbial community may increase the incidence of host. A previous study has indicated that the *Actinobacteria* content was noticeably increased in sheep diarrhea (26). Moreover, the synergy between *Actinobacteria* and host can influence pathogenic interactions in the intestine (35). Those results revealed distinct alterations in the relative richness of preponderant bacterial phyla of Tibetan sheep, which further implied its gut microbial alterations.

At the genus level, the percentage of *Ruminococcaceae, Ruminococcus, Lachnospiraceae, Coprococcus, Acetitomaculum, Olsenella, Oxobacter, Ruminiclostridium, Intestinimonas, Butyrivibrio, Pseudobutyrivibrio, Eubacterium_coprostanoligenes*, and *Prevotella* in *E. granulosus*-infected Tibetan sheep was obviously reduced as compared to control Tibetan sheep. *Ruminococcaceae*, a potential intestinal probiotic, is beneficial to degrade cellulose and starch and negatively correlated with liver cirrhosis and non-alcoholic fatty liver (36). It is reported that *Ruminococcus* plays a crucial role in degrading cellulose and produces small-chain fatty acid, e.g., formic, lactic, and acetic acids (37). Moreover, *Ruminiclostridium, Lachnospiraceae, Coprococcus, Acetitomaculum, Olsenella*, and *Oxobacter* can also produce short-chain fatty acids (38–42). Previous studies have revealed that short-chain fatty acid plays a key role in regulating gut microbial balance and maintaining the morphology and functionality of intestinal epithelial cells (43, 44). Lactic acid ameliorates digestive enzymes' activity and possesses bacteriostatic effects *via* regulating the gastrointestinal pH (30). Therefore, the higher abundances of *Ruminiclostridium, Ruminococcus, Lachnospiraceae, Coprococcus, Acetitomaculum, Olsenella*, and *Oxobacter* contribute to improve the growth and reduce gastrointestinal bacterial diseases in animals. It is known that *Intestinimonas* and *Pseudobutyrivibrio* produce butyrate and are essential for host health (45). Butyrate can decrease appetite and activate brown adipose tissue through the brain–gut axis, resulting in reducing cardiovascular disease and diabetes caused by obesity (46, 47). Currently, butyrate-producing bacteria are considered potential probiotics for treating and alleviating inflammatory bowel disease due to their anti-inflammatory and immunomodulatory functions (48). *Eubacterium_coprostanoligenes* is an anaerobe and possesses the ability to lower cholesterol (49). Remarkably, the abundance of *Eubacterium_coprostanoligenes* is negatively associated with the severity of anxiety (24). *Butyrivibrio* and *Prevotella* principally participate in the digestion and decomposition of cellulose and carbohydrate (50). Moreover, they can produce short-chain fatty acid (51). By contrast, the percentages of *Clostridium, Kocuria, Slackia, Achromobacter*, and *Stenotrophomonas* were significantly higher in infected Tibetan sheep. *Clostridiae* cause toxemia and diarrhea in ruminants (52). Moreover, it has been reported that *Clostridium* contributes to the occurrence of necrotic enteritis in infants (53). Previous studies have indicated that *Kocuria* can cause catheter-related bacteremia peritonitis in humans (54, 55) *Slackia* can lead to empyema and acute respiratory distress syndrome (56). Moreover, Shao and Zhu showed that the level of *Slackia* was dramatically increased in humans exposed to various metals for a long time (57). *Achromobacter* and *Stenotrophomonas* are the emerging pathogens, closely related to cystic fibrosis and bacteremia (58, 59). Our results revealed that *E. granulosus* infection could cause distinct dynamic changes in gut microbial community *via* increasing the proportion of pathogenic and beneficial bacteria. Previous research indicated that *E. granulosus* infections could impair intestinal mucosa and intestinal barrier function and alter intestinal mucosal immunity (60). Moreover, gut microbial community has also been demonstrated to play key roles in intestinal permeability and immune system maturation (61, 62). Thus, gut microbial dysbiosis may influence the immunity and intestinal barrier function, which in turn increases the risk for other diseases. Some opportunistic pathogens, such as bacteria, fungi, and viruses, are residing as part of normal gut microbiota but may take opportunity to result in diseases in gut microbial dysbiosis and immunocompromised situations (63, 64). Remarkably, this study also conveyed an important message that hydatidosis may be prevented through improving the quantity of beneficial bacteria in the intestine.

In conclusion, the present study investigated the influence of *E. granulosus* infection on the gut microbiota of Tibetan sheep. Results demonstrated that *E. granulosus* infection significantly altered the gut microbial composition, characterized by a decreased percentage of beneficial to pathogenic bacteria. Remarkably, there were several limitations in the present study, including a small sample size, individual variation, external environment, and failure to investigate the influence of intestinal fungal communities and viruses on *E. granulosus* infection.

## Data Availability Statement

The datasets presented in this study can be found in online repositories. The names of the repository/repositories and accession number(s) can be found below: https://www.ncbi.nlm.nih.gov/, PRJNA657975.

## Ethics Statement

The animal study was reviewed and approved by the Ethics Committee of the Anqing Normal University.

## Author Contributions

ZL conceived and designed the experiments. ZL and BY contributed to the sample collection, reagents, materials, and analysis tools. All authors contributed to the article and approved the submitted version.

## Funding

The Provincial Natural Science Research Project (no. KJ2017A357) at Anhui University, the Key Research and Development Plan of Anhui Province (no. 201904e01020013), the Science and Technology Development at Jilin Province, China (20180520040JH), and the Scientific Research Project of Education Department of Jilin Province (JJKH20210410KJ) supported this work through a planning grant program.

## Conflict of Interest

The authors declare that the research was conducted in the absence of any commercial or financial relationships that could be construed as a potential conflict of interest.

## Publisher's Note

All claims expressed in this article are solely those of the authors and do not necessarily represent those of their affiliated organizations, or those of the publisher, the editors and the reviewers. Any product that may be evaluated in this article, or claim that may be made by its manufacturer, is not guaranteed or endorsed by the publisher.
